# Risk Factors for Bovine Cysticercosis in North-West Italy: A Multi-Year Case-Control Study

**DOI:** 10.3390/ani11113049

**Published:** 2021-10-25

**Authors:** Selene Rubiola, Barbara Moroni, Luca Carisio, Luca Rossi, Francesco Chiesa, Giuseppe Martano, Elisa Cavallo, Luisa Rambozzi

**Affiliations:** 1 Department of Veterinary Sciences, University of Turin, Largo Paolo Braccini 2, 10095 Grugliasco, Italy; barbara.moroni@unito.it (B.M.); luca.rossi@unito.it (L.R.); francesco.chiesa@unito.it (F.C.); luisa.rambozzi@unito.it (L.R.); 2 Department of Agricultural, Forest and Food Sciences, University of Turin, Largo Paolo Braccini 2, 10095 Grugliasco, Italy; luca.carisio@unito.it; 3 ASL TO3, Animal Health, Via Martiri XXX Aprile 30, 10093 Collegno, Italy; gmartano@aslto3.piemonte.it (G.M.); ecavallo@aslto3.piemonte.it (E.C.)

**Keywords:** bovine cysticercosis, risk factors, *Taenia saginata*, *Cysticercus bovis*, case-control study, meat inspection

## Abstract

**Simple Summary:**

Bovine cysticercosis is a parasitic disease caused by *Cysticercus bovis*, the larval stage of the human tapeworm *Taenia saginata*, which is the causative agent of human taeniasis, a foodborne parasitic disease caused by the consumption of infected raw or undercooked beef meat. Although commonly asymptomatic, bovine cysticercosis has an important impact worldwide, causing huge economic losses to the meat industry due to carcass condemnation or downgrading. Given the lack of epidemiological case-control surveys carried out in Italy, the present survey’s aim was to assess the presence of farm-level risk factors for bovine cysticercosis in an endemic area in North-West Italy. The results of our survey show a significant association between the detection of bovine cysticercosis cases at slaughter and farm proximity to picnic spots, closeness of wastewater treatment plant effluents, presence of employees along with the family members and loose-housing systems. These findings highlight the need for enforced food chain information and surveillance strategies and the crucial role that hygiene-related campaigns would play to educate both the general public and farm operators on the transmission pathways of *T. saginata*.

**Abstract:**

*Taenia saginata* is the causative agent of bovine cysticercosis, a zoonotic parasitic disease with a worldwide distribution. Bovine cysticercosis is considered to be an important food safety issue responsible for human taeniasis and a major economic concern since infected carcasses undergo condemnation, freezing and downgrading. The aim of the current investigation was to assess the presence of farm-level risk factors for bovine cysticercosis in an endemic area in North-West Italy. A questionnaire was designed to collect information relating to several farm structural features, as well as management practices, environmental characteristics and attitudes of farmers. The questionnaire was administered in two separate time intervals by direct interview to previously selected case and control farms. Overall, 32 case farms and 131 control farms were included between 2005 and 2011 and 50 case farms and 192 control farms were included between 2014 and 2020. The present survey showed a significant association between the detection of bovine cysticercosis cases at slaughter and farm proximity to picnic spots, closeness of wastewater treatment plant effluents, loose-housing systems and presence of employees along with the family members, highlighting the need for targeted awareness raising policies.

## 1. Introduction

Bovine cysticercosis (BC) is a foodborne parasitic disease caused by *Cysticercus bovis*, the larval stage of the human tapeworm *Taenia saginata*. The two-host life cycle includes cattle as intermediate hosts and humans as definitive hosts. These latter become infected by eating raw or undercooked beef infected by viable cysticerci, which develop in sexually mature tapeworms three months after ingestion [1]. Proglottids, containing up to 150,000 eggs, are disseminated in the environment through feces, and can remain viable for several months [2]. Transmission to cattle can occur through the consumption of pasture, feed or water contaminated with eggs, although direct transmission of eggs resulting from handling of cattle by infected humans has also been rarely reported [3]. Once ingested, each egg may release an oncosphere which can penetrate the intestinal wall and travel via blood to striated muscles, developing as infective cysts. The viable cysticerci can be found mainly in masseter muscles, heart, tongue, diaphragm and other skeletal muscles, less commonly in fat or visceral organs [4]. After a period of time ranging from weeks to years, cysticerci undergo degeneration, frequently followed by calcification. Both viable and degenerated cysts may be found in the same carcass [5].

Human taeniasis (HT) is usually asymptomatic or characterized by mild gastrointestinal symptoms, such as nausea, vomiting, weight loss and diarrhea, sporadically associated to complications; although generally not considered as a health threat, the active migration of proglottids from the anus and the length of the tapeworm observed by the host when expelled can cause psychological stress, making it unacceptable in most European countries [6]. Bovine cysticercosis usually causes no symptoms, particularly if infections are light [7]. Nevertheless, BC has an important economic impact worldwide, causing huge economic losses to the meat industry due to the condemnation, freezing and downgrading of infected carcasses [8,9]. A review conducted in 1991 [10] estimated the value loss for a carcass due to BC at US $234/carcass and medical costs for taeniasis at US $111/patient in industrialized countries. More recently, the economic impact was estimated at an average economic cost of €3,408,455/year for each cattle owner in Belgium [11].

As taeniasis in humans is not notifiable in the European Union (EU), available data are scarce and incomplete, and its uncertain prevalence, estimated to range between 0.01% and 10% in EU, is usually predicted from the sale of anthelmintic drugs [12,13]. In Italy, the estimated prevalence ranges between 0.02% and 0.04% [7]. Although BC is reported worldwide, most of the available data are based on routine meat inspection reports, a detection method with low sensitivity. The estimated prevalence ranges from 0% up to 7.8% according to the European meat inspection data, but prevalence data based on serological and modeling methods ranges from 0.5% to 38.4%, highlighting an important underestimation of cases [7]. In Italy, BC prevalence data based on meat inspection are likely to range between 0.02% and 2.4%, but estimates may vary considerably between regions [14].

Notwithstanding the mild symptoms of human taeniasis and the underestimated low prevalence, meat hygiene globally has focused on this disease for centuries, treating it as a food safety issue and a huge economic concern [6]. Recently, European legislation regarding the meat hygiene inspection system has been revised to get as close as possible to the ideal risk-based, longitudinally integrated and flexible meat safety system [15]. Current controls are based on visual inspection, on the heart and the internal and external masseter incisions for cattle categories older than 8 months, or older than 20 months if reared without access to pasture during their w–hole life. Besides, in accordance with Article 30 of Regulation (EU) No. 2019/627, incision of the masseters at post-mortem inspection can be considered not compulsory by the competent authorities if: (A) a specific serological test is used, (B) the animals have been raised on a holding of provenance officially certified to be free of cysticercosis, or (C) the prevalence of the source population is below one in a million with 95% certainty, or no cases have been detected in all slaughtered animals in the past five years [16].

During official meat inspection procedures described above, the diagnosis of cysticercosis is based on the morphological appearance. Any lesion with fully transparent, cheesy or calcified cysts found in heart or masseter muscles is assumed to be a *Cysticercus* cyst [3,4]. Although meat inspection has a low sensitivity, estimated to range around 10–50% [3], a few false positive cases may occur, contributing to a small and usually negligible part of BC records [10]. Hence, the presence of abscesses, granulomas or parasites such as *Sarcocystis* spp. associated with eosinophilic myositis, may cause similar macroscopic lesions [17].

The recent revision of the European regulation has pointed out the need for enforced food chain information and surveillance strategies to implement the new risk-based, dynamic meat inspection strategies. Although several epidemiological surveys on BC, including risk factor studies, have been conducted in Europe, to our knowledge there is no data available about case-control risk factor studies carried out in Italy [2,18,19,20]. In this context, North-West Italy is well known for raw beef consumption, and can thereby be considered a high-risk area for BC. Besides, most of the farms in this area raise Piedmontese cattle, one of the most appreciated breeds on the Italian meat market; the important economic losses caused by condemnation, freezing and downgrading of infected carcasses are especially relevant for this cattle breed, known for the double-muscle phenotype, the superior meat quality and the low level of cholesterol. The know-how to address evidence-based prevention strategies of BC in endemic zones is thus essential [21].

In the attempt to contribute filling the aforementioned knowledge gap, we carried out a two-phase case-control study on farm-level risk factors for BC in a BC endemic area in Piedmont, North-West Italy.

## 2. Materials and Methods

The study area is located in the south-western part of the Province of Turin (Piedmont Region, North-West Italy), and includes approximately 550 km^2^ of plain and low mountains at the foot of the Alps. The centroid of the study area is located at 44°53′ N 7°20′ E, and the dense resident population exceeds 80,000 inhabitants (>145/km^2^). Cattle farms sum up to 946, of which 574 beef and 372 dairy farms, corresponding to a yearly average of 10,500 dairy and 11,500 beef cattle slaughtered in the investigated time intervals.

### 2.1. Selection of Case and Controls Farms

The definitions of case and control farms were based on recorded birth and movement data from the Italian National Bovine Database (BDN, Commission Decision 2006/132/EC) and individual slaughter records collected for cattle found to be infected with live or dead *Cysticercus bovis* during routine meat inspection at any of the EU-approved slaughterhouses located in the Province of Turin (North-West Italy) during the investigated time intervals. The diagnosis of BC was based on the morphological detection of viable or degenerated *T. saginata* cysticerci during meat inspection [16]. Case farms included all farms hosting at least one BC affected animal from birth until it was sent to slaughter and diagnosed with BC in any of two 7-year separated time intervals, between 1 January 2005 and 31 December 2011 and between 1 January 2014 and 31 December 2020, respectively. Control farms were randomly selected from the same EU-approved slaughterhouses within the same study area where case farms were recorded. Control farms did not host any BC case in the investigated time intervals. At least three control farms for each case farm were included in both studies (1:3 ratio of case to control farms) in order to achieve statistical power in later analyses [22].

### 2.2. Questionnaire Design and Administration

A questionnaire was designed to collect information relating to several farm structural features, as well as management practices, environmental characteristics and the attitudes of farmers. Putative farm-level risk factors were identified based on literature and the consultation of experts in the field of BC research [2,7,15,18,19].

The questionnaire was administered by direct interviews of case and control farm owners in July 2012 and January 2021 to collect information on 2005–2011 and 2014–2020, respectively. All interviews were conducted by the same person (M.G.) and administered to a responsible farm operator at the time of the on-site visit; the interviewer could thus verify the structural features included in the questionnaire. Written informed consent was provided to all participants before the interview.

The questionnaire was divided into nine parts and covered in total 62 questions on farmer’s identity and role on farm, farm and land management practices, including feed composition, origin and access, water systems, use of shared machinery, manure origin and use, presence of picnic spots near the pasture areas or sewage treatment plant in close proximity to the farm (3 km), people working in the farms and BC cases (Table 1). The interviews were conducted in Italian and all the questions were closed or semi-closed; notes fields were included in each section. Depending on the response of the interviewees, not all the farmers were asked all 62 questions; follow-up questions of no relevance according to the prior answer given were not asked. The majority of the questions were focused on those farm characteristics found to be associated with greater risk of BC according to the literature.

### 2.3. Data Analysis

Information related to farm features originating from the questionnaires was combined, cleaned and merged with data obtained from Italian National Bovine Database (BDN, Commission Decision 2006/132/EC).

A database including 25 variables was generated from the data gathered by the questionnaire, removing variables that were highly incomplete. Frequency tables were checked to identify and remove variables without level variation between positive/negative BC cases or with a low number of observations per variable level (<30) before statistical analysis. The BC prevalence was visualized to identify for which variables a meaningful effect on BC should be expected. The significant associations between a potential risk factor and the status of infected farm were analyzed using a multivariate generalized linear model (GLM), adopting a binomial error family and logistic link. Models and statistical analysis were performed using R 4.0.2 software [23]. Since the first model included all variables as fixed effects, the variance inflation factor (VIF) was calculated to identify collinearity among variables [24,25]. Variables having the highest VIF were dropped up to obtain a model whose variables had VIF < 7. Having removed collinearity, we identify the most explanatory model (lowest AIC) using the stepAIC function [26]. The best candidate model was used to calculate the marginal effect for significant risk factors (*p* < 0.05). 

Spatial analysis was performed using QGIS software 3.2.0 “Bonn” [27]. The geographic location of case and control farms was obtained by the Italian National Bovine Database (BDN).

## 3. Results

### 3.1. Descriptive Statistics

The criteria for the selection of the case and control groups resulted in the selection of 32 case farms and 131 control farms for the time interval 2005–2011, and 50 case farms and 192 control farms for the time interval 2014–2020. Most of the selected farms (51.11%) raised Piedmontese cattle, followed by Friesian cattle (25.93%) and mixed breed farms (22.96%), mostly housing French beef breeds.

Between 2005 and 2011, the presence of viable or degenerated *T. saginata* metacestodes was recorded in 128 cattle originating from 32 farms (12 dairy farms, 17 beef farms and three mixed farms). Between 2014 and 2020, BC was detected in 76 animals from 50 farms (13 dairy farms, 32 beef farms and five mixed farms). The distribution of the BC cases per case farm showed that only one animal with BC was detected in the majority of case farms in 2005–2011 (68.7%; 95% CI 49.99–83.88%) and 2014–2020 (78%; 95% CI 64.04–88.47%) respectively. Notably, in 2005–2011, 3 out of 32 case farms were home of massive outbreaks, referred to as “cysticercosis storms”, with 14, 21 and 40 individuals affected in few months.

**Figure 1:**
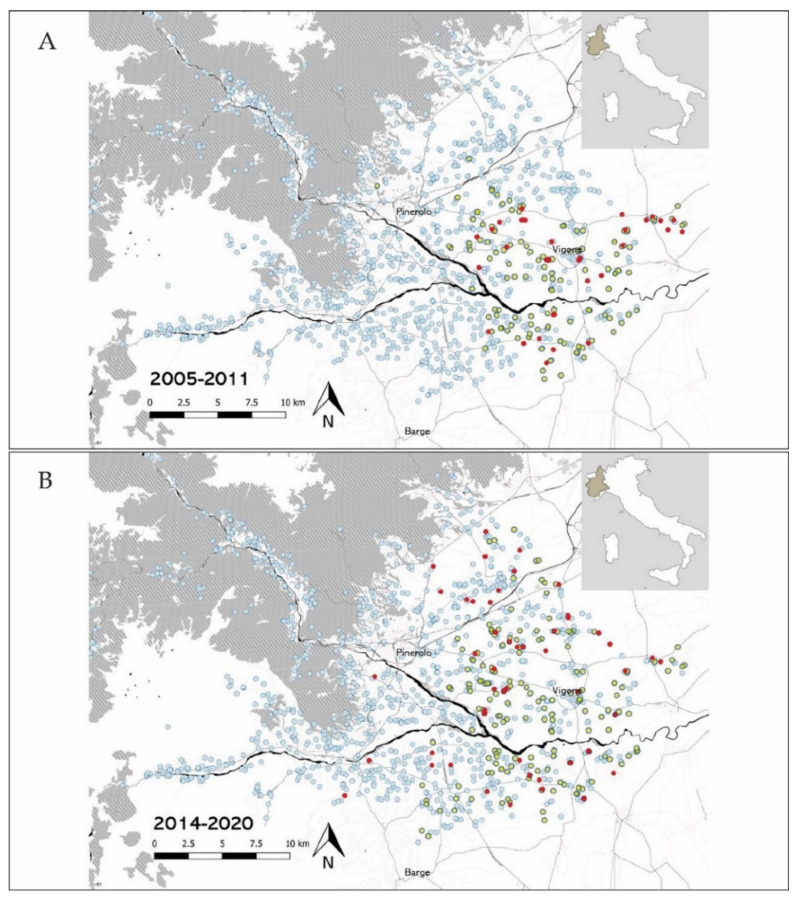
Distribution of case and control farms in the investigated area of Piedmont region. Red dots represent case farms detected between 2005 and 2011 (**A**) and between 2014 and 2020 (**B**), while control farms are represented by yellow dots. Empty light blue dots represent all farms recorded by the BDN in the southern part of the Province of Turin.

Overall, the median herd size was higher in case farms (130, range 22–791) than in control farms (79, range 16–541) (non-parametric equality-of-medians test *p* < 0.001). The distribution of case and control farms did not show evident clustering by visual inspection of the generated GIS maps (Figure 1). Forty-seven farms were surveyed in both case-control studies. Among these, forty-two resulted negative to BC in both surveys, four resulted positive in the first case-control study but not in the second one, one resulted positive in both surveys. BC prevalence rates per level of surveyed variables are shown in Supplementary Figure S1.

### 3.2. Risk Factors

Statistical analysis revealed 4 risk factors associated with a farm having at least a BC infected cattle (Table 2).

**Figure 2:**
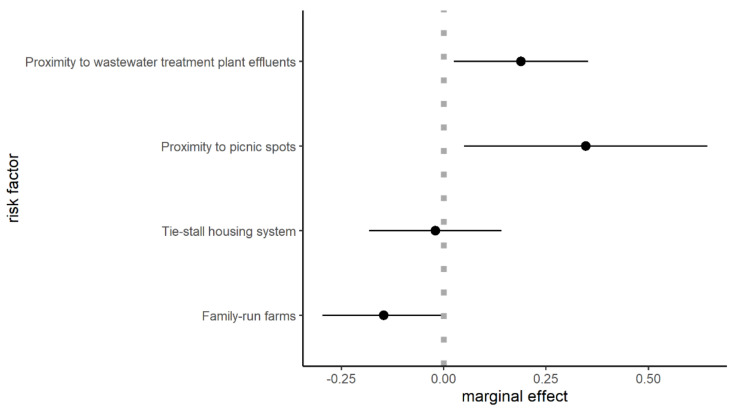
Estimated marginal effects for significant variables (*p* < 0.05). Dots indicate the average marginal effect while bars indicate standard error. Dotted lines indicate the threshold between negative/positive effect on the probability of BC infection.

Close-to-picnic-spots farms were 34% (*p* = 0.014) more likely to be infected than farms without picnic spots near or on farmland grazed by cattle; besides, the proximity to wastewater treatment plant effluents increased the probability of BC infection of 18% (*p* = 0.009). Presence of additional employees (maximum number recorded: 2) along with the family members and loose-housing systems also represented an increased risk: family only-run farms were 15% less likely to have BC cases (*p* = 0.027), while farms with tie-stall housing system had 16% lower (*p* = 0.024) probability of BC than farms with loose-housing system. Estimated marginal effects for significant variables are shown in Figure 2.

## 4. Discussion

Between 2005 and 2011 and between 2014 and 2020, 128 and 76 *T. saginata cysticercus* infected cattle were recorded in 32 and 50 farms located in the southwestern part of the Province of Turin, respectively. Through 14 years of official slaughterhouse data on BC, we identified a significant association between farms affected by BC and: (i) the proximity to leisure activities areas (picnic spots); (ii) the closeness of wastewater treatment plant effluents; (iii) the presence of employees along with the family members; (iv) the loose-housing system. Among the forty-seven farms that were included in both surveys, forty-two resulted negative in both time periods, four resulted positive only in the last survey and one resulted positive in both surveys. Interestingly, the latter farm did not show any change in farm management characteristics, while two over the four farms that were BC positive in the first survey but negative in the second survey showed changes in the farm management measures adopting lower-risk strategies: both converted the housing system from loose to tie-stall, and one farm prevented herd grazing in areas close to wastewater treatment plants.

The overall results of our study indicate a sporadic emergence of new cases of bovine cysticercosis in Northwestern Italy, which is in accordance with the low prevalence recorded in Europe in the last decades [5,17]. The higher number of BC cases recorded during the first case-control study was the result of the uncommon presence of three massive outbreaks, referred to as “cysticercosis storms”, characterized by the presence of up to 40 BC cases in affected farms. High infection rates of BC are relatively rare events, and mostly associated with proximity to sewage treatment plants [28] or uncontrolled human defecation in camping or picnic areas [29]. Interestingly, in the three positive farms, internal staff employees were diagnosed with taeniasis and some of them confessed having had unhygienic toiletry behaviors around the farm, although the results of the questionnaire did not identify the presence of taeniasis cases among farm operators as significant risk factors. *Taenia saginata* eggs can survive in the environment for several weeks or months [28], confirming the idea that the same infected person can pollute the environment and the water sewage for years, perhaps explaining the persistence of case farms in Piedmont region. In a control perspective, sanitary education of this particular category would be desirable.

The presence of picnic spots near or on farmland resulted in the most significant risk factor (*p* = 0.014) for case farms. Piedmont region is known for its tradition of raw or undercooked beef consumption [4], which is considered a common dietary habit increasing the prevalence of human taeniasis [3,7]. Accordingly, it is reasonable to assume that human taeniasis might be underestimated in this region and camping or picnic areas are at high risk of environmental contamination. Not only the consumption of raw beef, but also the high demographic pressure has been deemed as possible risk factor for a higher incidence of bovine cysticercosis [8,15].

The proximity of farms to wastewater treatment plant effluents as a significant risk factor associated to BC has been reported in the present investigation, as well as in previous studies [18,30], assuming that the most common transmission pathway might be through water sources contaminated with *T. saginata* eggs used by farmers for irrigation purposes. Contaminated water streams are known to favor a low-grade though diffuse and persistent BC infection rates [21].

Among farm characteristics, the loose-housing system has shown to be a significant risk factor for the presence of BC. In tie-stall housing systems each animal is tied up in a stall, where all routine activities take place, including resting, feeding, watering and management activities. On the other hand, in loose-housing systems cattle can move freely inside the farm shelter and most often between the shelter and an outside yard.

Whereas this housing system better gets over the welfare concerns about the limitations imposed by tie-stall, including restriction of movements and limitation of expression of natural behavior, data in this study suggest that loose-housing is a not negligible BC risk factor, as cattle have access to multiple spaces which might potentially be contaminated. This housing system is now adopted worldwide and is rapidly increasing together with the modernization process involving dairy and meat production systems, which includes the need to meet the growing interest of the consumers and the general public in animal welfare [31]. However, since shifting from a tie-stall to a loose housing system seems to imply a “cost” in terms of facilitated encounter with environmental *T. saginata* eggs, initiatives should be taken to raise the awareness of farmers operating in BC endemic areas, with a special focus on pathways leading to egg introduction in farm buildings.

Lastly, the presence of farm employees other than family members resulted in a significant risk factor for BC cases. Not surprisingly, however, the assumption that employees were more likely to be infected with *T. saginata* than family members was not supported by the HT cases recorded by the questionnaire. HT is still considered an infection whose diagnosis and eventual communication to third people is somehow embarrassing, hence it stands to reason that relevant information might have been omitted by interviewees, or they were simply unaware carriers of the parasite, as human taeniasis is frequently asymptomatic. Our findings highlight the importance of further investigating the potential human source of infection amongst farm operators by means of complementary and robust monitoring tools, including molecular coprological tests, especially in presence of massive BC outbreaks [1,32]. Bearing in mind that different monitoring tools are available, in addition to traditional coproscopy, high-throughput coproantigen tests and molecular coprological tests should be considered as robust and cost-effective monitoring tools [33].

## 5. Conclusions

In this study, different sources of data, such as meat inspection recordings, geographical coordinates of farms, national veterinary database and results from the farmer questionnaire, were collected to combine relevant epidemiological information on BC, highlighting the importance of an integrated approach to monitor parasitic infections of public health significance.

Limitations of the present study can be identified. First was the absence of data on the serological status of cattle in the case farms, which could have given additional information on the possible origin of the infection. The lack of any diagnostic data on HT among farmers and farm employees is also an obvious limitation in this and many similar studies. Finally, it cannot be excluded that the detection of BC based on meat inspection at the abattoir, a well-recognized “hugely insensitive detection method” [7,32], may have unintentionally resulted in some sort of bias; indeed, the possible presence of control farms which could have been classified as case farms applying a more sensitive BC detection method cannot be excluded. Nevertheless, most of the available data on BC are based on official meat inspection reports worldwide, stressing the fundamental role they actually play to perform epidemiological studies. Given the insufficient data on individual and farm-level risk factors of this important parasitosis in Italy, we encourage further epidemiological investigations aiming to enhance control surveillance systems in the areas known for a higher BC/HT risk. Other multi-sectorial intervention actions, such as hygiene and food related campaigns should be also conducted to educate the general public and bovine-specialized operators on the transmission pathways of *T. saginata*. Moreover, in a One Health perspective, the inclusion of physicians and humanities scientists should be considered as a value-added approach to train the target population, such as picnic area visitors and workers, as already outlined in other zoonotic parasitosis [34], about the life cycle of *T. saginata* and the risks of unhygienic toiletry, through the use of infographic material placed in identified areas.

## Figures and Tables

**Table 1:** Description of variables included in the risk analysis for BC in North-West Italy between 2005 and 2011 and between 2014 and 2020.

Variable	Description
Farm characteristics	Personal details of farmers; farming type (tie-stall or loose housing system *, herd replacement, grazing); herd size, sex ratio and breed of cattle.
Feed origin and composition	Roughage type; hay, silage, feed supplements and pasture percentage; hay, silage, feed supplements and pasture on-farm produced and/or purchased; grazing practices.
Water source and wastewater	Water supply system; presence of wastewater treatment plant effluent in proximity; sewer system type; presence of septic tank sludge; use of shared machinery.
Watering system	Flooding of pastures, source of water, season and rate.
Manure	Application of manure on pastures; manure source (on-farm produced/other farms); use of shared machinery.
Surrounding pastures	Leisure activities near or on farmland grazed by cattle (picnic spots).
Employees	Presence of employees along with the family members.
Taeniasis cases	Presence of employees diagnosed with taeniasis.

* Tie-stall housing systems: cattle is tied up in a stall, where all routine activities take place; loose-housing systems: cattle can move freely inside the farm shelter.

**Table 2:** Factors associated with farms found infected with bovine cysticercosis in North-West Italy.

Variable	Odds Ratio	95% CI	*p*-Value
Proximity to picnic spots	13.16	1.697–125.079	0.014
Proximity to wastewater treatment plant effluents	4.932	1.460–16.475	0.009
Family-run farms	0.267	0.082–0.877	0.027
Tie-stall housing system	0.843	0.229–3.566	0.024

## Data Availability

The datasets used and analyzed during the present study are included in the article. Raw data and further inquiries can be directed to the corresponding author.

## Supplementary Materials

The following are available online at https://www.mdpi.com/2076-2615/11/11/3049/s1, Figure S1: BC prevalence rates per level of surveyed variable. Variables not present in multivariate general model were discarded due to low number of observations per level or correlation with other variables.
