# Correction: Rubiola et al. Risk Factors for Bovine Cysticercosis in North-West Italy: A Multi-Year Case-Control Study. *Animals* 2021, *11*, 3049

**DOI:** 10.3390/ani12050636

**Published:** 2022-03-03

**Authors:** Selene Rubiola, Barbara Moroni, Luca Carisio, Luca Rossi, Francesco Chiesa, Giuseppe Martano, Elisa Cavallo, Luisa Rambozzi

**Affiliations:** 1 Department of Veterinary Sciences, University of Turin, Largo Paolo Braccini 2, 10095 Grugliasco, Italy; barbara.moroni@unito.it (B.M.); luca.rossi@unito.it (L.R.); francesco.chiesa@unito.it (F.C.); luisa.rambozzi@unito.it (L.R.); 2 Department of Agricultural, Forest and Food Sciences, University of Turin, Largo Paolo Braccini 2, 10095 Grugliasco, Italy; luca.carisio@unito.it; 3 ASL TO3, Animal Health, Via Martiri XXX Aprile 30, 10093 Collegno, Italy; gmartano@aslto3.piemonte.it (G.M.); ecavallo@aslto3.piemonte.it (E.C.)

Error in Institutional Review Board Statement [1]:

In the original publication, the Institutional Review Board Statement was incorrect. The authors apologize for the omission and for any inconvenience caused, and state that the scientific conclusions are unaffected. The original publication has also been updated.

Corrected Institutional Review Board Statement:

“The authors confirm that this study has been ethically reviewed by the Bio-Ethics Committee of the University of Turin [UOR: SI000045—Classif. III/11] under protocol number 0011298 and that ethical approval was granted in January 2012.”
